# High power electromagnetic pulse applicators for evaluation of biological effects induced by electromagnetic radiation waves

**DOI:** 10.1039/c8ra00330k

**Published:** 2018-05-01

**Authors:** Flavien Pillet, Laure Gibot, Alexandre Catrain, Jelena Kolosnjaj-Tabi, Kristelle Courtois, Thomas Chretiennot, Elisabeth Bellard, Jacques Tarayre, Muriel Golzio, René Vezinet, Marie-Pierre Rols

**Affiliations:** Institut de Pharmacologie et de Biologie Structurale, IPBS, Université de Toulouse, CNRS, UPS Toulouse France rols@ipbs.fr; CEA, DAM, GRAMAT F-46500 Gramat France

## Abstract

The effects of electromagnetic radiation waves on health is one of the major public concerns. These waves are mainly produced at a large scale but it is important to evaluate these effects on biological samples at the laboratory scale. Here we developed a set of micro applicators, which allow evaluating the effect of electromagnetic fields on biological samples with volumes in the microliter range. The applicators can be coupled to an optical microscope and allow a real-time observation of potential structural and functional alterations of the tested sample induced by different waveforms. New design approaches are suggested to simultaneously achieve maximized electric field coupling effect and optimized electric field homogeneity in the tested sample, while minimizing the return loss when the applicators are loaded with the biological samples. These applicators allow studying the biological effect of a variety of different signals, due to their wide frequency bandwidth (beyond 1.5 GHz) and their high permissible power. In addition, different electromagnetic parameters such as the electromagnetic field magnitude, pulse repetitive factor, number of bursts or delay between bursts may be set. The efficacy of the applicators was addressed for three different signals: two types of electromagnetic waves – a damped sinusoid centered at 200 MHz (wide band signal), a radar-like signal at 1.5 GHz (the ultra-narrow band signal) and a train of millisecond square-wave monopolar electric field pulses (causing electroporation). The biological effects were thus assessed (at the microscopic scale) on two different biological models, the giant unilamellar vesicles, and tumor and normal human cells, as well as being compared to results obtained (at full scale) with signals generated by antennas.

## Abstract 2

Micro applicators for real-time observation of electromagnetic radiation waves effects on giant unilamellar vesicles and mammalian cells.

## Graphical abstract

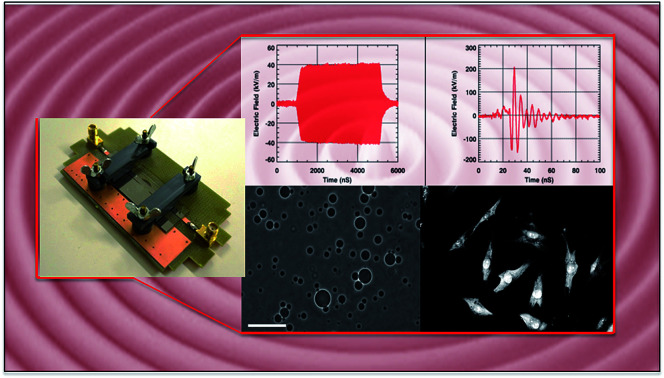

## Introduction

Electromagnetic radiation, which has natural and anthropogenic origins and is ubiquitous in our environment, can be emitted and absorbed, and its energy units, the photons or quanta, have an energy content equaling *E* = *hν*, where *h* is the Plank's constant and *ν* is the frequency. In our daily environment, visible light represents one of the major natural sources of electromagnetic radiation. While visible light does not represent a threat to human health because its energy is low (2 eV), the effects of electromagnetic radiation might be harmful (*e.g.* high-energy radiation above 5 keV, such as X-rays, may disrupt the covalent bonds within the molecules). The consequences of electromagnetic radiation thus depend on the radiation energy and the exposure time or the radiation dose.^1^

Although concerns have been raised over the radiation derived from modern electromagnetic radiation emitting devices such as mobile phones, radios, wireless devices, and radars, generated pulsed electromagnetic fields may also have beneficial effects, and thus can be applied to promote healing,^2,3^ improve antineoplastic therapies, stimulate the immune system,^4^*etc.* Detecting and understanding the effects of electromagnetic radiation would, for example, help in optimizing its use in biomedical fields, allow determining any potential adverse effects due to civilians' daily exposure to electromagnetic radiation emitting devices and help establish occupational health and workplace safety provisions for the members of armed forces.

With the aim to simulate the exposure to radio frequency sources, in order to study their effects on macromolecular and biological systems at a laboratory scale, we have developed submillimetric-sizes radiofrequency applicators and tested them on two different biological models of increasing complexity: giant unilamellar vesicles (GUVs) and living (normal and cancer) human cells. GUVs represent a useful basic and convenient model of a biological cell, allowing to mimic the behavior of the plasma membrane of cells submitted to electrical stress.^5^

Due to their size, comparable to mammalian cells, GUVs can be observed under a microscope and the effect of membrane deformation and permeabilization can be visualized. Imaging can be used in order to get a direct access to the underlying consequences of pulse application. Therefore GUVs allow addressing questions such as the effect of electric pulses parameters on membrane permeabilization and the associated shape alteration, due to the absence of cytoskeleton, as well as lipid loss resulting in a decrease in size of the permeabilized vesicles. Both human primary dermal fibroblasts obtained from a skin biopsy and a human colorectal cancer cell line were used, allowing to take into account different cell behavior under electric pulses applications.^6^ These models were chosen to evaluate potential direct and indirect effects on cell membrane and cell organelles, induced by short (nano- and micro-second-long) pulses, generated by bipolar oscillations. The applicators, which are intended to serve as surrogate systems for full-scale field experiments, generally performed in large hangars, are designed in a way that optimizes the electromagnetic field around the tested sample and applies a homogenous electromagnetic field throughout the tested sample. This issue is emphasized here because biological samples have a moderate to high relative permittivity, and therefore might cause strong impedance mismatch when placed in transmission line-based radiofrequency applicators. The direct consequence is that bigger tested samples can result in a higher loss of the reflected signal (also known as higher return loss). Different radiofrequency exposure setups applicable to *in vitro* studies were previously described,^7^ and they mainly include the waveguides,^8^ and transverse electromagnetic (TEM) cells, also known as Crawford cells.^9,10^ In addition, the use of radial transmission lines^11^ and radiofrequency chambers^12^ was also reported.^13^ Be that as it may, the best electromagnetic field homogeneity was reported for TEM cells, where biological samples are placed in Petri dishes, which are installed in the septum of the TEM cell. The drawbacks of this setting are that the gap between central and ground conductors of the TEM cell is filled with air and the tested biological sample occupies a small volume. As a result, the electric field coupling in the sample is low. Increasing the sample volume in the Petri dish may improve the electric field coupling in the tested sample, but this deteriorates the return loss.

New design approaches are suggested to simultaneously achieve maximized electric field coupling effect and optimized electric field homogeneity in the tested sample, while minimizing the return loss when the applicators are loaded with the biological samples. These applicators allow the testing of the effects of a variety of different signals, due to their wide frequency bandwidth (beyond 1.5 GHz) and their high permissible power. In addition, different electromagnetic parameters such as the electromagnetic field magnitude, pulse repetitive factor, number of bursts or delay between bursts may be set. Among different electromagnetic waves that can be used in the electronic warfare, high power ultra-narrow- and wide-band waves are emerging tools used for military and defense purposes to neutralize the opponents' electronic devices. While current legal guidelines provide norms applicable to different waveforms, these norms mainly focus on clearly defined thermal effects. Yet, while thermal effects might be predominant for radar-type waves, the occurrence of other (athermal) effects can not be excluded.

Here we present a set of micro applicators, in which giant unilamellar vesicles and living cells can be submitted to electromagnetic fields and which can be coupled to an optical microscope, in order to allow a real-time observation of potential structural alterations of the tested sample. We assessed potential biological effects of ultra-narrow band (UNB) waves and wideband (WB) waves to which the army staff and civilians could be exposed and their effects on GUVs and human cells were studied with the developed applicators on the laboratory scale. Finally, the results obtained with the applicators were compared to the results obtained in field experiments after exposure of tested material to real-size WB and UNB signals generated by antennas.

## Results and discussion

### Characteristics of the electromagnetic signals

**Fig. 1:**
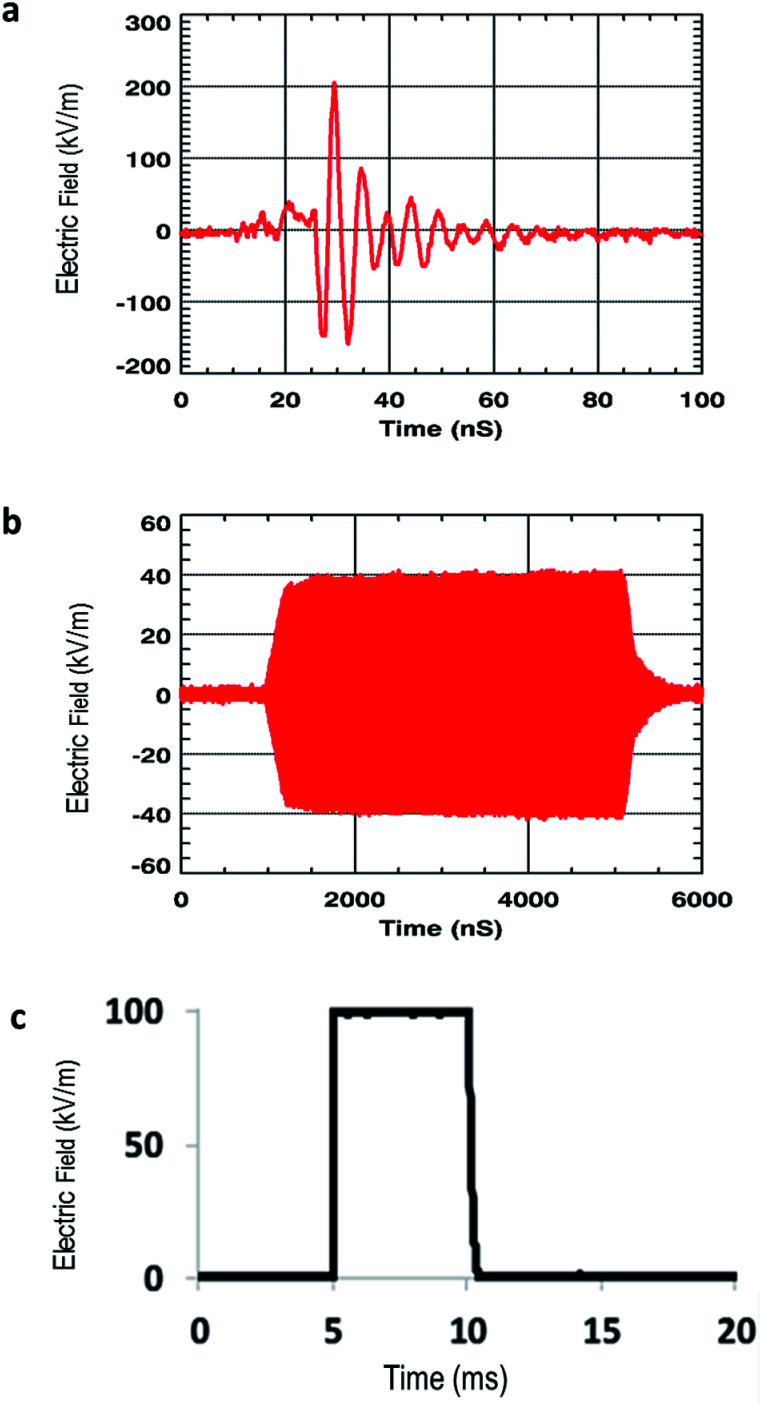
Waveforms (measured signals) to which GUVs and cells were submitted: (a) wideband (WB) waves, (b) ultra-narrow (UNB) waves and (c) pulsed electric fields.

Two different signals (Fig. 1a and b) were used. The electromagnetic waves radiated by antennas varied in terms of oscillating frequency, pulse duration, amplitude, repetition rate, polarity, damping and shape. Wideband waves (Fig. 1a) were applied at a 200 MHz frequency with a 200 kV m^−1^ amplitude radiated waves. Pulses duration was 20 ns, and 2500 pulses were applied. The shape of the signal is shown in Fig. 1a. Ultra-narrow band waves (Fig. 1b) were applied at a 1.5 GHz frequency with a 40 kV m^−1^ amplitude. Pulses duration was 4 μs and 50 000 pulses were applied. The shape of the signal is shown in Fig. 1b. As a positive control for the potential effects of electromagnetic fields on membranes and cells, we used square wave electric pulses (Fig. 1c), with parameters known to induce athermal transient permeabilization of GUVs and cellular membranes. Signal specifications and pulse exposures are reported in Table 1.

**Table 1:** Summary of the characteristics of applied pulses

Parameters	Wideband	Ultra-narrow band	Electroporation
Frequency	200 MHz	1.5 GHz	None
Frequency of repetition	100 Hz	1 kHz	1 Hz
Pulse duration	20 ns	4 μs	5 ms
Number of pulses	2500	50 000	10
Max incident electric field radiated by antennas or applied between electrodes	200 kV m^−1^	40 kV m^−1^	1 kV cm^−1^
Application time	25 s	50 s	10 s
Polarity	Bipolar	Bipolar	Unipolar
Shape	Pseudo sinusoidal	Sinusoidal	Square wave
Damping	Damped	Undamped	Undamped
Time domain waveform	Fig. 1a	Fig. 1b	Fig. 1c

### Design of sub-millimetric sizes applicators

**Fig. 2:**
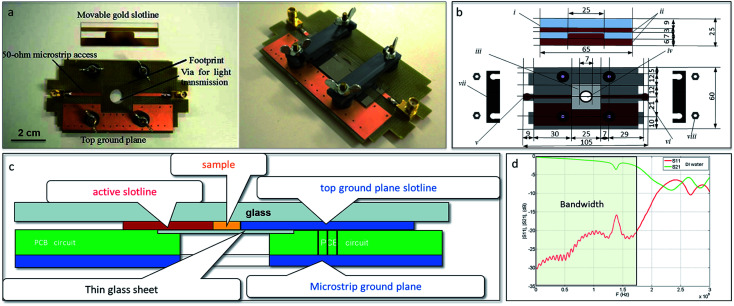
Applicator for high power pulsed electromagnetic field exposure for GUVs/living cells. (a) Photograph of the disassembled (left) and assembled (right) device (length approx. 8 cm), (b) the schematic representation from the top plane of the disassembled device including (i) a movable glass micro-machined slide, (ii) gold electrodes, (iii) glass coverslip onto which the sample is placed, (iv) circular orifice that allows the visualization of the tested sample by a light microscope, (v) radiofrequency input, (vi) radiofrequency output, (vii) movable bolt, (viii) clamp, and (c) applicator's simplified view from the transversal plane through the 50 ohm microstrip at the sample position. On the fixed board of the device, metallic *via* holes enable the microstrip ground to be reported on the top face and thus connect the ground of the slotline. The GUV/cell suspension sample is exposed to the microwave pulses between the two conductors of the slotline. The gap between the two conductors is 250 μm. According to the small surfaces of these chamber electrodes (200 μm × 2.5 cm), the parallel impedance perturbation does not affect the 50 ohms microstrip behavior at low frequencies (measured |S11| ≤ −10 dB from 0 to 1.8 GHz). (d) Electric response (return loss |S11| and insertion loss |S21| parameters) of the applicator loaded with deionized water.

Sub-millimetric sizes applicators for high power pulsed electromagnetic fields exposure were inspired from Krishnaswamy *et al.*^14^ and adapted in order to allow studying the effects of electromagnetic fields on GUVs or cell suspensions.^15^ Briefly, the applicators (Fig. 2) are constituted of two 50 ohm-microstrip lines etched on a polychlorinated biphenyl (PCB) circuit, which is fixed on the stage of a microscope. A hole at the center of the PCB allows light transmission from the light source (Fig. 2a left panel). A glass coverslip is placed in a footprint, etched throughout the thickness of the board. Prior to observations, GUVs and cells are deposited on this glass coverslip placed in the footprint of the applicator. The cell suspension is then covered with the movable slotline, which is screwed on the top face of the board and shunts the 50 ohm-microstrip line (Fig. 2b right panel). The schematic views of the device are represented in Fig. 2b and c. The GUVs or cells are thus stranded between the two electrodes of the slotline, the thin glass sheet at the bottom and the insulating substrate of the movable slotline, and can thus be exposed to electromagnetic fields induced by wideband (WB) waves, ultra-narrow (UNB) waves, and pulsed electric fields. The pulses propagate between the two conductors of the slotline. The gap between the conductors is 250 μm. The applicator was experimentally validated with samples of deionized (DI) water, and Fig. 2d shows return and insertion losses (IS11I and IS21I respectively).

The applicator allows submitting GUVs and cells to peak field intensities of 200 kV m^−1^ (after WB exposure at 200 MHz) and 40 kV m^−1^ (after UNB exposure at 1.5 GHz) after 100 V and 20 V voltage supply, with a bandwidth from −3 dB to 1.7 GHz and a maximal field homogeneity of 99%, as evaluated with full wave simulations. As GUV/cell suspensions are in tight contact with the electrodes (as the sample fills all the space between the conductors), the electric field coupling is maximized.

### Effect of pulsed electric fields on giant unilamellar vesicles

Cellular membranes are one of the first barriers the waves encounter, and might potentially interact with. The worst-case scenario of wave–membrane interactions could include effects such as membrane deformations, loss of transversal symmetry, permeabilization and fusion of two adjacent membranes.^16^ The GUVs represent a model lipid bilayer, which was used in the first-tier test to assess the membrane response to WB and UNB pulses.

**Fig. 3:**
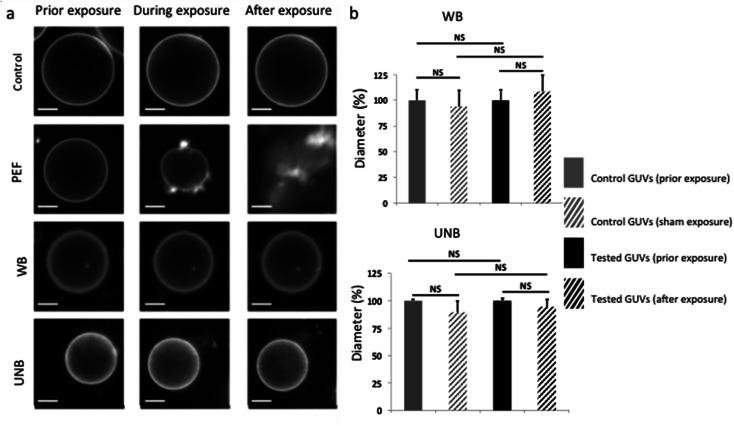
Effects of the electric field exposure on GUVs assessed within the applicator (a) representative fluorescence microscopy micrographs of control GUVs and GUVs submitted to pulsed electric field (PEF) with 10 pulses of 5 ms at 1 kV cm^−1^ and a frequency of 1 Hz, wideband (WB) and ultra-narrow band (UNB) pulsed electromagnetic field. The micrographs were obtained 10 seconds prior exposure, during the exposure and 10 seconds after the exposure (scale bar 10 μm). (b) Influence of WB and UNB on the size distribution of GUVs measured prior exposure and 10 seconds after WB and UNB exposure (*N* = 30 GUVs per condition) (NS = no statistical significance).

As expected and illustrated in Fig. 3a, the application of pulsed electric fields (PEFs) at 1 kV cm^−1^ induces irreversible membrane electroporation associated to lipid loss and GUV destruction. This is a classical phenomenon occurring during electroporation,^17^ used as positive control, and demonstrates the applicators capacity to efficiently transmit desired electric fields. We thus proceeded to an array of tests in order to assess a potential lipid loss from the GUVs after application of pulsed WB or UNB electromagnetic fields. If GUVs loose lipids, their diameter decreases. As depicted by histograms on Fig. 3b, we did not observe any significant GUVs diameter decrease, which could correlate with a potential athermic effect related to WB pulse exposure (10 Hz, 2500 pulses) or UNB pulse exposure (100 Hz, 50 000 pulses).

**Fig. 4:**
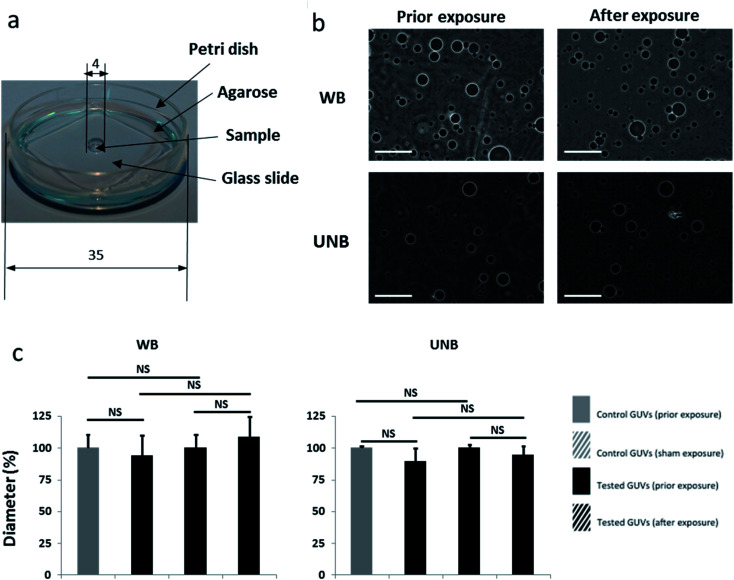
Field experiment setting and results after GUVs exposure to signals generated by antennas generating high power WB and UNB signals (systems DIEHL and TEMPETE). The experiments were performed at the French alternative energies and atomic energy commission (CEA) of Gramat, France (a) the Petri dish (*d* = 35 mm) containing the sample and 1 mL of agarose (2%) resulting in a 1 mm thick layer covering the dish bottom. The sample is placed in the 4 mm well within the gel and covered by a thin glass slide. (b) Phase contrast micrographs showing GUVs prior and 15 minutes after exposure to WB and UNB pulses (scale bar 50 μm). (c) Influence of WB and UNB on the size distribution of GUVs measured prior exposure and 15 minutes after WB and UNB exposure (*N* = 400 GUVs per condition), showing no size effects related to WB or UNB exposure.

We then compared GUVs exposed in free space to WB and UNB electromagnetic waves radiated by real-size antennas (Fig. 4). Wide band EM wave was generated by the build in dipole antenna of the commercial DIEHL DST110T high power system. Ultra narrow band EM wave was generated by a 100 cm in diameter commercial parabolic antenna, which is part of the commercial GERAC TEMPETE high power system.

A homemade device in agarose Petri dish was thus designed (Fig. 4a). This geometrical configuration allows to minimize the volume of the sample, to maximize the *E* field coupling factor and to maximize its homogeneity in the sample volume.^18^ The coupled field into the sample at the center of the Petri dish (Fig. 4a) was numerically evaluated and its value was 48% of the incident radiated field.^18^ Consequently, we have limited the applied fields within the laboratory applicators to these values. For each condition tested, 400 GUVs were observed by phase contrast microscopy before and after exposure (Fig. 4b). After WB and UNB exposure, we did not observe any statistical GUVs diameter variation (Fig. 4c). As expected, results after exposure to real-size antennas were similar to submillimetric-sizes radiofrequency applicators.

### Effect of pulsed electric fields on tumor and normal human cells

**Fig. 5:**
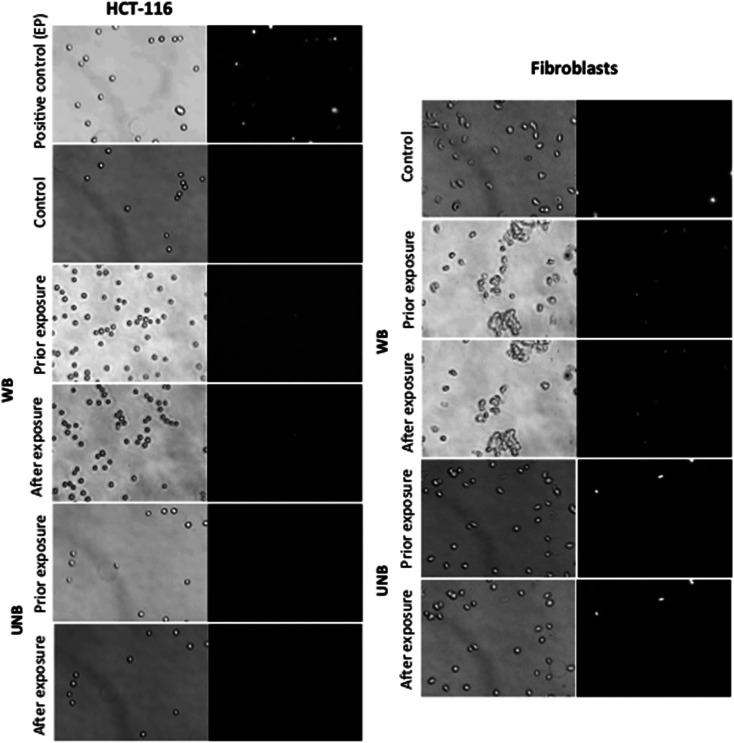
Effects of electromagnetic waves on plasma membrane permeabilization on tumor (HCT-116) and normal human cells (fibroblasts): bright field (left of the column) and fluorescence micrographs (right of the column) of tumor (HCT-116) and normal cells (fibroblasts), acquired with a wide-field fluorescence DMIRB Leica microscope coupled to a Photometrics Cool SNAP HQ camera (exposure time = 100 ms). From top to bottom: micrograph obtained after cells submission to 1 kV cm^−1^, 100 μs duration, 8 pulses at 1 Hz frequency pulsed electric field, control cells non submitted to electric fields, and cells prior and after WB and UNB pulses exposure, in presence of 100 μM of propidium iodide. Note that the absence of fluorescent signal after pulsation correlates with an intact (non-permeabilized) cell membrane. (Mag ×20, pulsing conditions WB: *F*_1(sinusoid)_ = 200 MHz, *F*_2(modulation)_ = 100 Hz, 1.19 kV cm^−1^, 2500 pulses; UNB: *F*_1_ = 1 kHz, *F*_2_ = 1.5 GHz, 0.4 kV cm^−1^, 100 000 pulses).

In order to address a potential response of healthy and cancerous cells, which proliferate at a higher rate and exhibit enhanced metabolic processes, the effect of WB and UNB pulses were assessed on human dermal fibroblasts and human colon cancer cell line (HCT-116) (Fig. 5). Cells in suspension were placed in the applicator together with the propidium iodide, which was used as a probe for membrane permeabilization detection.^19^ As this device did not allow retrieving the cells after their exposure, the experiments consisted in assessing the morphology of the cells and the putative plasma membrane permeabilization. As expected and shown in Fig. 5, the application of pulsed electric fields (PEFs) at 1 kV cm^−1^, 100 μs, 8 pulses at 1 Hz frequency induces the permeabilization of all the cell that became fluorescent. In contrast, no impact on the cell membrane in terms of morphology (such as an increase in size or blebs apparition) and in terms of permeability was detected for both cell types when exposed to electromagnetic fields under our experimental conditions, even when the most severe experimental conditions were applied (WB: 100 Hz, 2500 pulses; UNB: 1 kHz, 100 000 pulses).

**Fig. 6:**
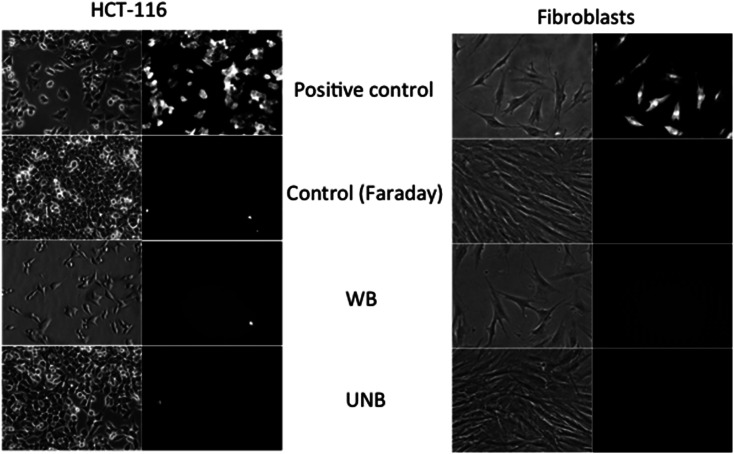
Field experiment settings and results after adherent cells exposure to signals generated by high power antennas generating WB and UNB signals (systems DIEHL and TEMPETE). The experiments were performed at the French alternative energies and atomic energy commission (CEA) of Gramat, France. Bright field and fluorescence micrographs of tumor (HCT-116) and normal cells (fibroblasts), acquired with a wide-field fluorescence DMIRB Leica microscope coupled to a Photometrics Cool SNAP HQ camera (exposure time = 100 ms). From top to bottom: micrograph obtained after cells submission to, 1 kV cm^−1^, 100 μs duration, 8 pulses at 1 Hz frequency pulsed electric field, control cells stored in a Faraday cage close to the antennas, and cells after WB and UNB pulses exposure, in presence of 100 μM of propidium iodide. Note that the absence of fluorescent signal after pulsation correlates with an intact (non-permeabilized) cell membrane. (Mag ×10, pulsing conditions WB: *F*_1(sinusoid)_ = 200 MHz, *F*_2(modulation)_ = 100 Hz, 1.19 kV cm^−1^, 2500 pulses; UNB: *F*_1_ = 1 kHz, *F*_2_ = 1.5 GHz, 0.4 kV cm^−1^, 100 000 pulses).

The results obtained with applicators were then compared to field experiments obtained after cells exposure to real-size antennas. The obtained results are shown on Fig. 6. The cells grown on Petri dishes, were exposed to PEFs (used as a positive control) and electromagnetics fields (WB and UNB) together with propidium iodide, and then further observed under a microscope. For each condition, a minimum of 300 cells were visualized. As expected, the application of PEFs induces the permeabilization of both cancer and healthy cells as clearly shown by the uptake of the fluorescent dye. All the cells being fluorescent, the efficiency of permeabilization is 100%. In contrast, and in agreement with the results obtained in the laboratory with the applicators, no effect of WB nor UNB could be detected in terms of permeability or fusion. The very rare fluorescent cells corresponded to already dead cells.

## Conclusions

In this study we describe the design of radiofrequency applicators, where submillimetric electrode circuit allows the study of the exposure of minimal volumes of GUVs or living cell suspensions to radiofrequency wave pulses. In the present work we sought to examine the non-thermal effect of two waveforms: a damped sinusoid centered at 200 MHz the wideband (WB) signal, and a radar-like ultra-narrow band (UNB) signal at 1.5 GHz. The applicators, coupled to a light microscope, enabled us to study the morphological effects of electromagnetic waves on GUVs and cells (size and shape), which, if altered, could attest to some harmful effects of tested waveforms. Under our experimental conditions, the applied WB and UNB pulses did not induce any observable changes to the macromolecular or cellular samples tested. In contrast, when the parameters of electromagnetic pulses were set to values, which are known to cause membrane damage, the lipid bilayers visibly changed their structure, and the cell membranes became permeabilized.^19^ The applicators are designed in a way that optimizes the electromagnetic field around the tested sample and applies a homogenous electromagnetic field, which is measured *in situ* in the close vicinity of the tested sample. New design approaches are applied to achieve a maximized electric field coupling effect and optimize the electric field homogeneity in the tested sample, while minimizing the return loss when the applicators are loaded with the biological samples. In addition, these applicators offer the possibility to tune electromagnetic parameters such as the electromagnetic field magnitude, pulse repetitive factor (PRF), number of bursts or delay between bursts and are thus appropriate to study the effect of different electromagnetic fields exposures on the laboratory scale. Finally, the similar results observed with the real-size antennas validated the efficiency of the submillimetric applicators to expose biological samples at the lab scale.

In conclusion, we developed a set of micro applicators, which allow evaluating the effect of electromagnetic fields on biological samples with volumes in the microliter range. The applicators can be coupled to an optical microscope and allow a real-time observation of potential structural and functional alterations of the tested sample induced by different waveforms. We validate the design of radiofrequency applicators, on GUVs and living cell suspensions. Under the conditions used in this study, neither wide band nor narrow band signals induce any observable effects on the biological membranes and cells. Nevertheless, the presented devices could indeed be useful to investigate other higher power signals, and find the limit where effects might appear. Moreover, these sets-up could be used by the scientific community to study other radiofrequency wave pulses.

## Experimental

### Device design

The designed wide-band applicators are developed to allow the assessment of the biological effect of two waveforms: a damped sinusoid centered at 200 MHz the wideband (WB) signal, and a radar-like ultra-narrow band (UNB) signal at 1.5 GHz. The coupled electric field homogeneity in the tested sample is evaluated with a 3D electromagnetic simulation and assessed with the following formula: 1 − (*E*_max_ − *E*_min_)/*E*_max_, where *E*_max_ and *E*_min_ are the maximum and minimum electric field strengths in the tested sample. Electromagnetic simulations that preceded the creation of the device, had been made with CST Microwave Studio – 3D electromagnetic simulation software, a commercially available full-wave field solver, based on the Finite Difference Time Domain (FDTD) method. These applicators were designed by the CEA Gramat.

The device structure and the fabrication of the electrodes have been adapted from Sun *et al.*^24^ The following material and structural features were applied: (i) a transparent chamber to visualize the real time effects of electromagnetic and electric fields, (ii) the dimension of the chamber was adapted to tested models (GUV or cells), (iii) gold electrodes were used as a noncorroding conductive surface to avoid potential artefacts, (iv) SU-8 was used to build up the walls of the chamber. The applicator for high power pulsed electromagnetic fields exposure, which was used in our study, consists of an assembly (Fig. 2), made of a movable glass micro-machined slide including a 30 μm thick resin layer fabricated by photolithography, the 200 μm thick gold electrodes spaced 0.25 mm apart (for WB or UNB signal, respectively, because smaller inter-electrode distance correlates with stronger electric field).

**Fig. 7:**
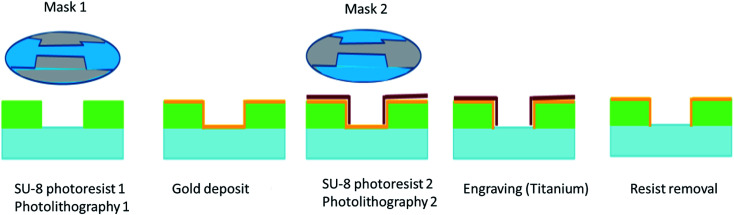
Process of electrodes fabrication by photolithography and engraving.

A schematic representation of the different steps of fabrication is depicted in Fig. 7.

The glass of the slide acts as an insulator, while the electrodes make an electrical circuit when the movable slide and the support are mechanically assembled. The support is made of PCB and a 50 Ω transmission stripline.^20^ The movable glass slides were custom made by the Renatech platform of LAAS/CNRS institute (Toulouse, France), which followed our specifications for design. The sample under test (*V* = 250 μL) is placed on the glass coverslip as shown on Fig. 2. The applied voltage at the sample position can be easily monitored with an oscilloscope used as a 50 ohm matched termination load through a high voltage pulse attenuator (BARTH Electronics).

The High Tension Unipolar (HTU) generator (β-tech, Saint-Orens-de-Gameville, France) is used to generate the pulsed electric fields (Fig. 1c); the microwave generator Agilent 81150A (Agilent, Santa Clara, USA) is used to generate WB waves centered at 200 MHz (Fig. 1a); the Agilent 81150A generator, coupled to a Marconi 2024 synthesized signal generator (Marconi Instruments/IFR, now Aeroflex; Plainfield, USA) are used to generate the UNB signal centered at 1.5 GHz (Fig. 1b). The generation of WB signal is obtained after signal amplification with the AR500A2501 amplifier (500 watts, 10 kHz to 250 MHz) and the generation of the UNB signal is obtained with a HF TMD-PTC amplifier, which can deliver tunable pulses with varying pulse amplitude (*U*) from 0 V to 2 kV, pulsation time (*t*) from 5 μs to 50 ns, period of repetition (*P*) from 1 ms to 10 s and number of repetitions (*R*) from 0 to 10 000. The electric field amplitude (*E*) is proportional to the voltage (*U*) between the two electrodes, and can be calculated by the formula *E* = *U*/*d*, where *d* is the distance between the electrodes. The oscilloscope (Agilent DSO9254A, Agilent, Santa Clara, USA), coupled to a radio-frequency attenuator (29 dB gain), allows the visualization of the input and the output voltage signal before and after the passage throughout the applicator. The electric setup included the use of BNC, SMA and N-type coaxial cables and N-SMA, BNC-SMA and N-BNC transitions. Experimental validations were confirmed on samples containing deionized water, which has similar electrical properties to biological samples (GUVs and tumoral/normal cells suspended in an aqueous milieu). The list of the full equipment needed for setting up the bench is provided below in Table 2.

**Table 2:** Summary of the list and characteristics of equipment

Equipment	Wideband	Ultra-narrow band	Electroporation
Generator	Agilent 81150A	Agilent 81150A coupled to a Marconi 2024 synthetized signal generator (10 kHz; 5.4 GHz)	BetaTech
Amplifier	AR500A2501 500 watts 10 kHz to 250 MHz	AF HF TMD-PTC	None
Attenuator	N type	N type	None
Oscilloscope	Agilent DSO9254A	Agilent DSO9254A	Agilent DSO9254A
Coaxial cables	BNC, SMA & N	BNC, SMA & N	BNC
Transitions	N/SMA, BNC/SMA & N/BNC	N/SMA, BNC/SMA & N/BNC	BNC

### Illumination in field experiments using Petri dishes

GUV field exposures have been performed using a Petri dish (*d* = 35 mm) containing the sample and 1 mL of agarose (2%) resulting in a 1 mm thick layer covering the dish bottom. The sample was placed in the 4 mm well within the gel and covered by a thin glass slide. In order to increase the *E* field coupling factor and to improve the homogeneity of the *E* field into the volume of a small DUT (Device Under Test, *i.e.* sample), an artificial extension of the volume of the DUT was made, using a dielectric material characterized by the same dielectric properties. The so-called FHVE extension (Field Homogenization by Volume Extension) aims at avoiding permittivity discontinuities at the DUT boundaries.

The Petri dish was placed in front of antennas of the WB and UNB high power microwave systems; the *H* orientation was chosen; in such configuration the *H* field component was axially oriented and the *E* field component was coplanar with the agarose layer.^18^ This orientation allows a coupling factor of 48% of the *E* field with the entire diameter dimension of the agarose layer and the homogeneity of the *E* field inside the sample volume, located at the center of the Petri dish is excellent due to similar dielectric properties of the agarose and the sample (DUT).^18^ This method for preparing an object to be tested and for improving the uniformity and intensity of an electric field induced in said object illuminated by an incident electromagnetic wave is related to a patent (Pat., 20170184648, 2015, Publication date: 2017, Applicant: Commissariat à L'energie Atomique et aux Energies Alternatives, Inventors: R. Vezinet, A. Catrain, T. Chretiennot). The thickness of the agarose layer has to be limited to 1 mm with the aim to avoid some dielectrics ringing modes in *H* orientation; see ref. 18 for details. The incident *E* field was measured at the Petri position and we have used this measured signal as an excitation for a 3D simulation within CST software using a plane wave illumination of the modeled Petri device; the dielectric properties of the sample and the agarose have been previously measured on the entire frequency spectrum of interest. The coupled *E*-field homogeneity in the DUT attained 72% and was evaluated with 3D electromagnetic simulation and assessed by the following formula: 1 − (*E*_max_ − *E*_min_)/*E*_max_ where *E*_max_ and *E*_min_ are the maximum and minimum *E* field strength in the DUT. Electromagnetic simulations have all been made with CST Microwave Studio, a commercial full-wave field solver based on the Finite Difference Time Domain (FDTD) method.

### WB and UNB signals

WB signals are representative from an HPM system like the DS-110 (built in suitcase) sale by the Diehl BGT Defence company in Germany. UNB signals are representative from EM wave radiated by defence radars. The experimental system TEMPETE sale by the GERAC company makes use of high power magnetrons as generators. The chosen amplitudes for the signals are related to the maximum *E* field witch can be radiated in near zone, in front of the antennas, in case of accidental exposition of technical people.

**Fig. 8:**
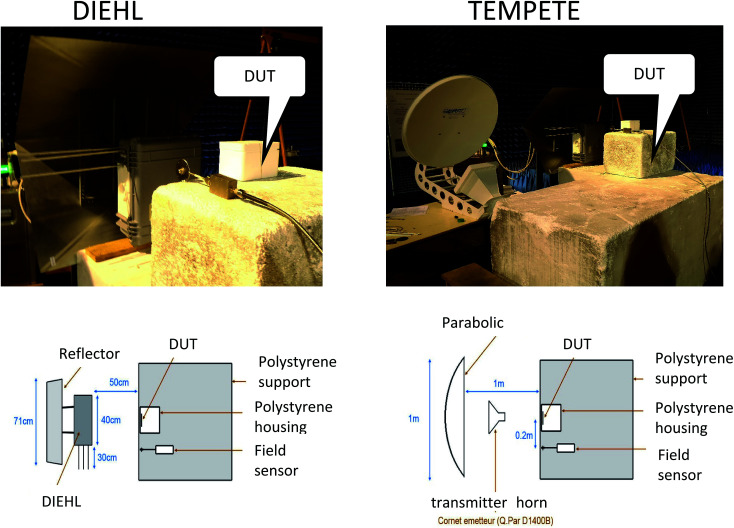
Description of the antennas experimental set-up.

These signals, reported in Fig. 1, are measured signals representative of the incident electric field at the level of the Petri dish. As shown in Fig. 8, an electric field sensor was installed during the experiments, and was placed at equal distance from the antenna and from the sample, either in the same axis, or at a position that was laterally shifted for 20 cm. A comparative measurement prior to the illumination of the sample confirmed the negligible effect of the shift on the amplitude of the incident field. Nevertheless, the field induced in the samples should take into account the coupling factor of 48%, which is stated further within the manuscript text. In the case of laboratory experiments, which were made using the device shown in Fig. 2, these signals correspond to the electric field applied between the two electrodes with a coupling factor of 100% and deduced from the measurement voltage 50 ohm at the end of the line (*E* (V m^−1^) = *V* (V)/*d* (m)) (*via* an attenuator).

Photo of these antennas and scheme showing the entire description of the set-up are given in Fig. 8.

### Giant unilamellar vesicles (GUVs)

The synthesis of GUVs followed the previously described protocol.^5^ The GUVs were formed from 1,2-dioleoyl-*sn-glycero*-3-phosphocholine (DOPC) lipids (99%) and the fluorescent, nitrobenzoxadiazole-labeled 1-palmitoyl-2-oleoyl-*sn-glycero*-3-phosphoethanolamine (NBD-POPE) lipid (1%).

### Cultured cells

Human colon carcinoma HCT-116 cell line was purchased from ATCC (catalog number #CCL-247). Primary normal human dermal fibroblasts obtained from a skin biopsy provided by Icelltis (Toulouse, France) were isolated as previously described.^21,22^ Both tumor and normal cells were cultivated in the DMEM culture medium (Gibco, Invitrogen, France) supplemented with 10% heat-inactivated fetal calf serum (Gibco, Invitrogen, France) and 100 μg mL^−1^ penicillin and 100 μg mL^−1^ streptomycin. Cells were cultured at 37 °C in a humidified atmosphere containing 5% CO_2_. Both cell types were tested negative for mycoplasma using MycoAlert mycoplasma detection kit (Lonza #LT07-318, USA).

### Plasma membrane permeabilization detection

The integrity of the plasma membrane was assessed using a small fluorescent probe, the propidium iodide (Sigma-Aldrich #P4170, Germany). Propidium iodide is a non-permeant DNA intercalant, which becomes highly fluorescent when intercalated in DNA of cells presenting plasma membrane defects. Briefly, during exposure to the radiofrequency signal, the cells were incubated with 100 μM propidium iodide diluted in PBS at room temperature. Red fluorescence was detected with a wide-field fluorescence DMIRB Leica microscope coupled to a Photometrics Cool SNAP HQ camera (Roper Scientific, Tucson, USA) after 100 ms exposure time. As positive control, cells were efficiently electropermeabilized (1000 V cm^−1^, 100 μs, ×8, 1 Hz) in the presence of 100 μM propidium iodide as previously described.^23^ Micrographs were normalized according to the positive control (*i.e.* electroporated cells). This treatment allowed qualitative assertion of fluorescence levels between distinct experimental conditions.

### Statistical analyses

At least six replicates were analyzed for each set of experiments. Data are expressed as mean ± SEM and overall statistical significance was set at *p* ≤ 0.005.

## Author contribution

F. P., L. G., A. C., K. C., T. C., E. B., J. T., M. G. performed the experiments, F. P., L. G., J. K. T., E. B., M. G., R. V., M. P. R. analyzed the data and contributed to the writing of the paper. R. V. and M. P. R. designed, coordinated and supervised the experiments.

## Conflicts of interest

There are no conflicts to declare.

## Supplementary Material
